# Anti-synthetase syndrome in a child with pneumomediastinum: a case report and literature review

**DOI:** 10.1186/s12890-024-02984-0

**Published:** 2024-04-01

**Authors:** Jieqiong Lin, Yaowen Li, Qimeng Fan, Longwei Sun, Weisheng Sun, Xin Zhao, Hongwu Zeng

**Affiliations:** 1 Department of Radiology, Shenzhen Children’s Hospital, Shenzhen, Guangdong Province 515038 China; 2 Department of Pediatric Intensive Care Unit, Shenzhen Children’s Hospital, Shenzhen, Guangdong Province China; 3 Shantou University Medical College, Shantou, Guangdong Province China; 4 China Medical University, Shenyang, Liaoning Province China

**Keywords:** Anti-synthetase syndrome, Mycoplasma pneumoniae, Pneumomediastinum, Children, Computerized tomography

## Abstract

**Background:**

Anti-synthetase syndrome (ASS) is a group of rare clinical subtypes within inflammatory myopathies, predominantly affecting adult females. Instances of critical illness associated with ASS in children are even rarer.

**Case presentation:**

We report the case of a 7-year-old boy finally diagnosed with ASS, combined with pneumomediastinum. He presented with intermittent fever persisting for 12 days, paroxysmal cough for 11 days, chest pain, and shortness of breath for 4 days, prompting admission to our hospital. Pre-admission chest CT revealed diffuse pneumomediastinum, subcutaneous pneumatosis in the neck and bilateral chest wall, consolidation, atelectasis, and reticular nodular shadowing in both lungs, as well as pericardial effusion and bilateral pleural effusions. Laboratory tests revealed a positive result for serum MP immunoglobulin M (MP-IgM) and MP immunoglobulin G (MP-IgG). The patient was initially diagnosed with mycoplasma pneumoniae (MP) infection, and following 3 days of antibiotic treatment, the patient's tachypnea worsened. Positive results in muscle enzyme antibody tests included anti–PL-12 antibody IgG, anti–Jo-1 antibody IgG, and anti–RO-52 antibody IgG. Ultrasonography detected moderate effusions in the right shoulder, bilateral elbow, and knee joints. Corticosteroids pulse therapy was initiated on the 27th day following disease onset, and continued for 3 days, followed by sequential therapy for an additional 12 days. The child was discharged on the 43rd day, and subsequent follow-up revealed a significant improvement in consolidation and interstitial lesions in both lungs.

**Conclusions:**

ASS in children may combine with rapidly progressive interstitial lung disease (RPILD) and pneumomediastinum. It is crucial to promptly identify concurrent immunologic abnormalities during the outbreak of MP, particularly when the disease exhibits rapid progression with ineffective conventional antibiotic therapy.

## Background

Anti-synthetase syndrome (ASS) is an autoimmune disorder characterized by inflammatory myopathies, arthritis, and cutaneous manifestations such as Raynaud’s phenomenon and Mechanic’s hands, along with interstitial lung disease (ILD) [1, 2]. ASS is one of the most prevalent subtypes of idiopathic inflammatory myopathies (IIMs), with distinctive serological markers being anti-tRNA synthetase antibodies (ARSs), including anti–Jo-1-ARS, anti–PL-7, anti–PL-12, anti-EJ, anti-KS, anti-Zo, anti-Tyr/YRS, and anti-OJ antibodies. Among these, anti–Jo-1-ARS is the most frequently observed in ASS patients [3].

When the lungs are affected, ASS manifests as nonspecific interstitial pneumonia characterized by diffuse ground-glass opacities and peripheral consolidation. Additional features encompass linear opacities, a honeycomb pattern, and traction bronchiectasis [4]. Pathological observations have revealed varying degrees of inflammation and fibrosis in the lung interstitium. Microscopically, this is characterized by infiltration of lymphocytes and plasma cells in the alveolar septa, which comprises collagen fibers exhibiting varying degrees of fibrosis mixed with chronic inflammation [5].

This study presents the case of an ASS child complicated with pneumomediastinum. The report underscores the importance for clinicians to pay attention to immunologic abnormalities when encountering a general infection that rapidly progresses to multi-system abnormalities during the outbreak of MP. Additionally, we conducted a literature review on ASS combined with pneumomediastinum, with the aim of enhancing understanding of ASS during infected states and preventing delays in treatment.

## Case presentation

A 7-year-old male was admitted to our hospital exhibiting intermittent fever persisting for 12 days (with a peak temperature of 39.0 °C), paroxysmal cough ongoing for 11 days, and chest pain along with shortness of breath for the past 4 days. Before admission, the patient had undergone antibiotic treatment at other medical facilities, however, it proved ineffective. At the time of admission to our hospital, his body temperature was 36.9℃, respiratory rate was 65 breaths/min, pulse rate was 64 beats/min, and oxygen saturation was 98.0% with mask oxygenation. The patient had obvious symptoms of hypoxia, including tachypnea, three-concave sign, rough and decreased respiratory sounds in both lungs. There were rales in the left lung. Subcutaneous crepitation was detected in the neck. No hemorrhages or rashes were observed on the skin and mucous membranes throughout the body. The patient exhibited normal muscle strength and tension. There was no reported history of abnormal development, allergies, surgery, trauma, family medical background, or chronic infectious diseases.

Chest radiography conducted prior to admission revealed diffuse consolidation of both lungs, pneumomediastinum, and subcutaneous emphysema. To further elucidate the extent of pneumomediastinum and characterize the lung lesions, a chest CT examination was performed on the patient. Chest CT disclosed subcutaneous pneumatosis in the neck and bilateral chest wall, pneumomediastinum, interstitial emphysema in both lungs, diffuse consolidation, reticular nodular shadowing in both lungs, atelectasis in the upper lobes of both lungs, and in the lower lobe of the left lung, in addition to pericardial effusion and bilateral pleural effusions. Laboratory tests revealed a positive result for serum MP immunoglobulin M (MP-IgM) and immunoglobulin G (MP-IgG). Metagenomic next-generation sequencing (mNGS) of bronchoalveolar lavage fluid indicated the presence of MP, Human gammaherpesvirus 4, and human metapneumovirus (Table 1). Tests for *Streptococcus pneumoniae* and group A streptococcus antigens returned negative results. PCR results for EV71 + CA16 + enterovirus and COVID-19 nucleic acid were also negative. Other relevant laboratory tests are detailed in Table 2. Noninvasive assisted ventilation was administered initially, followed by anti-infection medication (intravenous cefoperazone sulbactam and erythromycin) and anti-inflammation treatment (intravenous methylprednisolone, 2 mg/kg/day, b.i.d.).

**Table 1:** Metagenomic next generation sequencing(mNGS) result of bronchoalveolar lavage fluid

Pathogen/Species	confidence level	Sequence
Mycoplasma pneumoniae	99%	20,575
Human gammaherpesvirus 4	99%	8
Human metapneumovirus	99%	258

**Table 2:** Laboratory Data of the Patient

Time of illness, day	3	5	7	9	12	13	15	16	23	30	36	39	43
CRP, mg/L (0–10)	15.1	20.58	7.73	15.73	2.6	0.6	2.1	0.4	< 0.2	< 0.2	< 0.2	0.3	< 0.2
N, 10^9^/L (1.6–7.8)	3.99	5.49	7.17	7.38	10.91	14.12	11.65	12.48	10.3	12.71	23.95	4.63	5.88
WBC, 10^9^/L (4.3–11.3)	5.68	8.09	9.92	8.3	12.93	19.36	13.13	14.73	11.98	16.84	25.67	12.5	12.59
RBC, 10^12^/L (4.2–5.7)	4.35	4.77	4.53	4.31	4.52	5	4.66	4.67	4.19	4.4	4.34	4.41	4.56
Hb, g/L (118–156)	124	140	130	124	127	140	132	131	119	128	127	129	135
PLT, 10^9^/L (167–453)	274	307	481	446	675	892	758	746	483	475	442	412	453
PCT, ng/ml (< 0.05)	-	-	-	0.1	-	0.038	-	-	-	-	-	-	-
ESR, mm/h (0–20)	-	-	-	41	-	-	-	-	-	11	7	-	23
CK, IU/L (35–223)	-	-	-	-	-	21		22	18	86	27	-	-
CK-MB, ng/ml (0.79–4.13)	-	-	-	-	-	0.8		1	1.11	1.84	2.7	-	-
LDH, IU/L (192–321)	-	-	-	-	-	333	-	249	265	318	399	-	-
KL-6, U/ml (105–401)	-	-	-	-	-	-	-	-	1023	-	-	-	-

On day 13 of illness, due to evident shortness of breath, the child was transferred to the PICU, where he underwent antibody testing to help identify autoimmune disease as CT indicated interstitial lesions in both lungs. The autoantibody profile, encompassing 11 items (serum), revealed anti–Jo-1 antibody (+ +). Myositis antibody spectrum analysis showed anti–PL-12 antibody IgG ( +), anti–Jo-1 antibody IgG ( +), and anti–RO-52 antibody IgG ( +). We did not perform tests on antibodies to melanoma differentiation-associated gene 5 (MDA5) because of restricted examination scope. Sputum culture showed no abnormalities.

On day 18, after a slight stabilization of his condition, the child was transferred to the Department of Respiratory Medicine. Although the child did not have obvious joint swelling or pain, based on the positive results of several myositis antibodies, he was considered to have an autoimmune disease, and was recommended to undergo a joint examination to confirm the joint involvement. Ultrasound revealed synovial bursa effusion in the right shoulder joint, bilateral elbow joints, and knee joints.

**Fig. 1:**
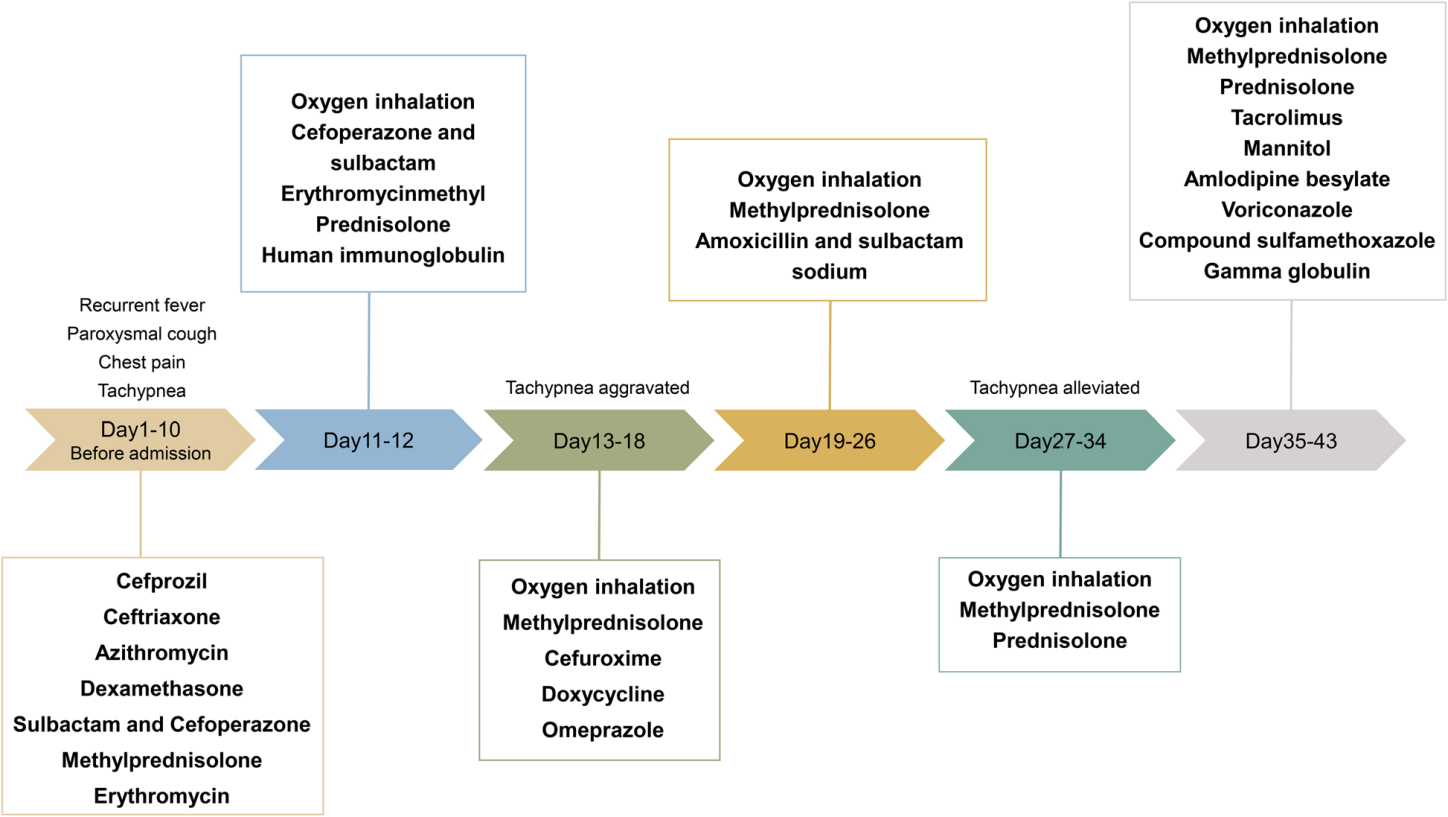
Clinical treatment course of the patient

On day 26, following a multidisciplinary consultation and discussion, ASS was considered. Pulse treatment (methylprednisolone, 500 mg/day, intravenously guttae, q.d.), was administered for 3 days, followed by sequential oral prednisolone (30 mg/day, q.d.) on days 30–34, which was then reduced to 25 mg/day on day 35. On day 35, oral tacrolimus (1 mg, q12H) was added (Fig. 1).

**Fig. 2:**
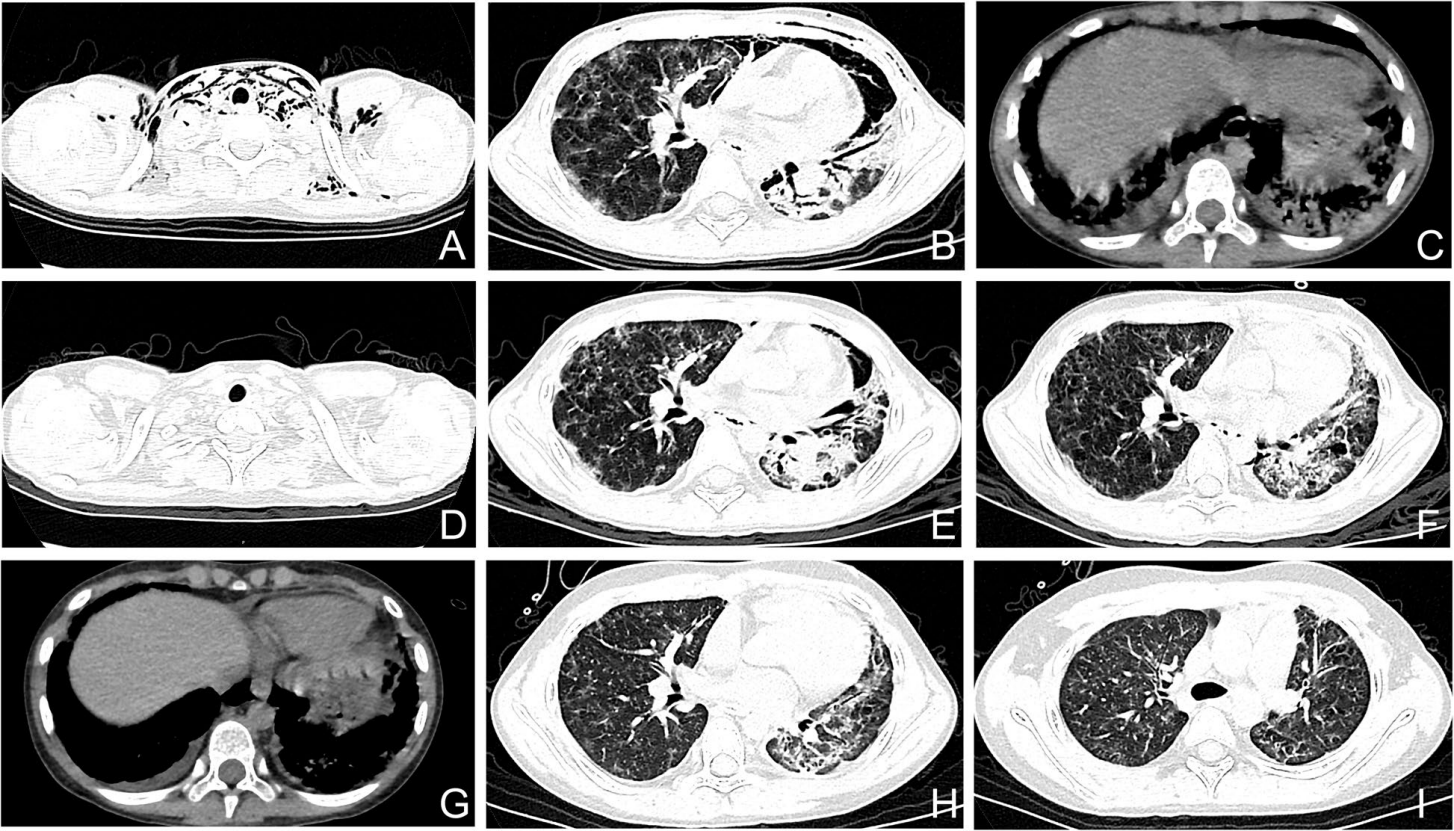
Radiologic findings of the patient. **A-C** On day 18 of illness, CT shows subcutaneous pneumatosis of the neck and bilateral chest wall, pneumomediastinum, consolidation, and reticular nodular shadow in both lungs, along with pericardial effusion and bilateral pleural effusion. **D-E** On day 23, subcutaneous air is essentially absorbed, pneumomediastinum is reduced compared with earlier, and consolidation and interstitial lesions in both lungs are present. **F-G** On day 30, pneumomediastinum is fully absorbed, and consolidation and pleural effusion are less pronounced than earlier. **H-I** One week after discharge, CT shows reticular nodular shadow, and both lungs exhibit residual consolidation, which have significantly resolved

Chest radiographs were reexamined on day 42, revealing the absorption of pneumomediastinum, bilateral cervical and axillary subcutaneous pneumoperitoneum, reduction of lesions in both lungs, and nearly complete absorption of left pleural effusion. Following treatment, the child's condition stabilized, and he was discharged from the hospital. At the time of discharge, he showed no signs of fever, cough, shortness of breath, wheezing, or cyanosis, and the pneumomediastinum had resolved. One-week post-discharge, the chest CT scan revealed residual interstitial lesions and consolidation in both lungs, indicating improvement compared to the CT scan on day 18 of illness (Fig. 2). During a follow-up visit 1 month later, inflammatory indexes showed no abnormalities. The patient received oral administration of tacrolimus and prednisone acetate tablets, along with fluticasone propionate inhaler prescription. Regular follow-ups were scheduled in the 3rd, 4th, and 5th months to monitor the blood concentration of tacrolimus, adjust the tacrolimus dosage, lower the prednisone dosage, and undergo regular hospital admission for gammaglobulin transfusion (1 g/kg) for immunotherapy regulation.

## Discussion

Pneumomediastinum, characterized by the entry of air into the mediastinum through connective tissue spaces within the pleura [6, 7], can be asymptomatic with a small gas accumulation. However, sudden onset or a significant influx of air compressing the organs within the mediastinum can result in respiratory and circulatory disorders, and in severe cases, be life-threatening.

The current case represents the first report of anti-synthetase syndrome in a child combined with pneumomediastinum. Previous studies revealed the similar phenotype of ASS in juveniles and adults, although this subtype is much less frequent in childhood than in adulthood. Important features such as Raynaud phenomenon, mechanics hands and ILD seem to occur at a lower frequency in childhood-onset disease than in adult-onset disease [8].

Our retrospective analysis of ASS cases combined with pneumomediastinum, previously reported in adults (Table 3), strongly indicates a correlation of ASS with ILD and pulmonary infection [9–11]. This underscores the pivotal role of infection in precipitating severe complications of ASS-associated ILD. In our case report, pneumomediastinum was potentially associated with ASS-associated rapidly progressive ILD (RPILD), with infection being a crucial factor in its progression. Remarkably, the MP and other virus infection are not the trigger for the onset of ASS. The patient in this particular case had an underlying preexisting condition of ASS, which had not been previously identified.

**Table 3:** Cases with anti-synthetase syndrome and pneumomediastinum

Reference	Age/sex	Primary disease	symptoms	Imaging	Drug Treatment	Intubation	Outcome	Diagnosis
Saint-Georges [9]	63Y/Female	Anti-synthetase syndrome	Fever; muscle ache; dyspnea; cough; facial papules-erythema predominantly on the eyelids	Extensive stripe shadow in both lungs; diffuse ground glass opacity; pneumomediastinum; pneumothorax	Corticosteroids; human immunoglobulin; immunosuppressants	Yes	Died	Anti-synthetase syndrome; pneumomediastinum; pneumonia; pulmonary fibrosis; acute respiratory failure; hypoxemia
Vinicki [10]	50Y/male	Hypothyroidism; type 2 diabetes; hypertension	Intermittent fever, weight loss, generalized muscle weakness, progressive dyspnea, facial erythema, scaly papules on the fingers	Pulmonary interstitial involvement with little ground glass, pneumomediastium, subcutaneous emphysema	Methylprednisone, azathioprine	No	Recovered	Anti-synthetase syndrome; pneumomediastinum; hypothyroidism; type 2 diabetes; hypertension
Jhajj [8]	48Y/male	Diabetic	Cough, low-grade fever, erythroderma over the ears and radial aspect of the fingers, weight loss, arthralgia	Patchy multifocal areas of peripheral airspace disease in the lungs with moderate fatty liver infiltration, pneumomediastium, subcutaneous emphysema	Prednisone, azathioprine, mycophenolate, hydroxychloroquine, rituximab, double lung transplant, tacrolimus	Yes	Recovered	Anti-synthetase syndrome; pneumomediastinum

Metagenomic next-generation sequencing (mNGS) of bronchoalveolar lavage fluid revealed the presence of MP, Human gammaherpesvirus 4, and human metapneumovirus. Notably, the number of sequences detected for MP was significantly higher than that for other pathogens. Considering the clinical manifestations in children, MP infection remains a significant factor to consider when explaining the rapid progression of interstitial lung disease; however, it is important not to exclude the possibility of infection with other viruses.

Numerous studies have confirmed that abnormalities in the immune mechanism play a significant role in the development of ASS-ILD, with a focus on the production of anti-synthesis enzyme antigen–antibody interactions [12, 13]. In vitro experiments have shown the presence of histidyl-tRNA synthetase (anti–Jo-1)–reactive CD4 + T cells in bronchoalveolar lavage fluid, indicating immune activation against anti–Jo-1 antibody in the lungs of ASS patients [14]. Antigen stimulation triggers innate and adaptive immune responses, leading to the production of antibodies that can be transferred to various tissues in the body.

Pneumomediastinum and subcutaneous pneumatosis are commonly observed in IIM patients with ILD [5, 7]. The mechanism underlying pneumomediastinum in IIMs remains unclear, with some scholars proposing that it may be induced by vasculitis and pulmonary fibrosis [7, 15]. Vasculitis can lead to necrosis of the alveolar and bronchial walls, disrupting the mucosal barrier and allowing air to enter the mediastinum. Pulmonary fibrosis, on the other hand, may result in lung atelectasis near the mediastinum, forming pulmonary bullae; rupture of these bullae can puncture the pleura, enabling air to enter the mediastinum and causing pneumomediastinum. Moreover, rapidly progressing lung infections, such as MP, can cause mucosal damage to alveolar walls, resulting in pneumomediastinum and pneumothorax.

Approximately 33% [16] of IIM patients can develop RPILD. ASS, accounting for 7.8% of IIMs, is associated with ILD in > 90% of cases [17], and ILD significantly contributes to increased prevalence and mortality in these patients [17, 18], and ASS patients with ILD often experience more severe disease with rapid progression. ILD associated with IIMs exhibit a high ICU admission rate of 26.7% and a mortality rate of 28.9% [16], emphasizing the importance of early diagnosis and aggressive treatment to prevent serious outcomes.

In the current case, the child did not exhibit significant manifestations of myositis; however, despite the absence of cutaneous features of connective tissue disease, myositis is not present in approximately 53% of ASS patients [2]. Positive results for anti–PL-12, anti–Jo-1, and anti–Ro-52 antibodies were reported in this case. Studies have shown that anti–Ro-52 antibodies in ASS patients are associated with RPILD [19], whereas anti–PL-12 antibodies are strongly associated with ILD development [20]. In a previous study, arthritis was the most common initial symptom in the anti–Jo-1 antibody-positive group [21], and in alignment with literature, our patient presented with multiple joint effusions. Additionally, this case highlights the importance of promptly considering the possibility of combined autoimmune-associated nonspecific ILD and, if necessary, conducting tests for IIM-associated antibodies when evaluating rapidly progressing infections with uncommon extrapulmonary complications.

## Conclusion

The occurrence of ASS in children is uncommon, and when infection is present, the disease may progress rapidly or even lead to pneumomediastinum. In the context of an epidemic of MP, if a child demonstrates rapid progression and uncommon extrapulmonary complications, pediatricians should consider the possibility of immune-related diseases and promptly conduct tests for relevant antibodies.

## Data Availability

The datasets used and analyzed during the current study are available from the corresponding author on reasonable request.
